# Serum klotho levels and mortality patterns in frail individuals: unraveling the u-shaped association

**DOI:** 10.1007/s40520-024-02730-w

**Published:** 2024-04-11

**Authors:** Huanhuan Luo, Zitian Zheng, Huixiu Hu, Chao Sun

**Affiliations:** 1 Department of Nursing, National Center of Gerontology, Institute of Geriatric Medicine, Beijing Hospital, Chinese Academy of Medical Science, NO.1 Da Hua Road, DongDan, Beijing, 100730 China; 2 Graduate School of Peking, Union Medical College, Beijing, People’s Republic of China; 3 Department of Orthopedics, Institute of Geriatric Medicine, Beijing Hospital, National Center of Gerontology, Chinese Academy of Medical Sciences, Beijing, People’s Republic of China; 4 Fifth School of Clinical Medicine, Peking University, Beijing, People’s Republic of China; 5 Department of Sports Medicine, Peking University Third Hospital, Institute of Sports Medicine of Peking University; Beijing Key Laboratory of Sports Injuries; Engineering Research Center of Sports Trauma Treatment Technology and Devices, Ministry of Education, Beijing, China

**Keywords:** Serum α-klotho level, Frailty, Mortality, NHANES

## Abstract

**Background:**

Frailty, a clinical syndrome intricately linked with the aging process, stands as a harbinger of numerous adverse outcomes, most notably mortality. This study aimed to elucidate the association between serum α-klotho concentration and mortality patterns, including all-cause and cause-specific mortality, in patients with frailty.

**Methods:**

The study employed Cox proportional hazard models, smoothed curve fitting, and supplementary analyses, encompassing threshold effect analysis, subgroup and sensitivity analyses, to explore the relationship between α-klotho levels and mortality, including all-cause, CVD, and cancer-related mortality.

**Results:**

Among the 2,608 frail individuals (mean age: 60.78 [SD 10.48] years; 59.89% female), the mortality stood at 25.35% during a median follow-up period of 6.95 years. Both unadjusted and adjusted models revealed a significant inverse association between higher serum α-klotho levels and the risk of all-cause and CVD-related mortality ([mean(95% CI) 0.68 (0.55, 0.83)] for all-cause mortality; [mean(95% CI) 0.48 (0.32, 0.74)] for CVD-related mortality, all P for trend < 0.001). Notably, log2–klotho displayed a U-shaped correlation with all-cause mortality and cancer mortality, characterized by thresholds of 9.48 and 9.55, respectively. The robustness of these findings was consistently supported by subgroup and sensitivity analyses.

**Conclusion:**

This study unveils a U shaped association between serum α-klotho levels and both all-cause and cancer-related mortality among middle-aged and elderly individuals with frailty in the United States. The identified serum α-klotho thresholds, at 714.8 pg/ml for all-cause mortality and 750.6 pg/ml for cancer-related mortality, hold promise as potential targets for interventions aimed at mitigating the risks of premature death and cancer within this vulnerable population.

**Supplementary Information:**

The online version contains supplementary material available at 10.1007/s40520-024-02730-w.

## Background

Frailty, an age-related clinical syndrome, is characterized by diminishing physiological reserve capacity, heightened vulnerability to stressors, and an elevated risk of adverse outcomes [1, 2]. As the global population ages rapidly, frailty has assumed critical significance in the realm of older adult health. Extensive meta-analyses have revealed frailty prevalence rates among older adults ranging from 10% to 51.5% across diverse settings [3, 4]. It is often intertwined with the decline in the physiological functioning of multiple organ systems, significantly amplifying the likelihood of adverse health consequences, particularly mortality [5].

Recent attention has been fervently directed towards unraveling the molecular underpinnings of frailty and exploring interventions that could ameliorate its detrimental impacts on health and longevity [6]. One such intriguing molecular candidate is Klotho, a protein endowed with diverse biological functions that has been intricately linked to the processes of aging and age-related ailments [7, 8]. Klotho includes three isoforms: α-, β-, and ⋎-Klotho, with the latter two identified through their homology with α-Klotho [7, 9]. Our study focuses on the α-klotho protein, consisting of five exons [10]. Considering its pertinence to the aging process, it stands out among the three isoforms and has been researched in this study.Initially renowned for its role in mineral metabolism and renal function, α-klotho has emerged as a potential regulator of the aging trajectory and age-associated maladies [9]. This single-pass transmembrane protein predominantly finds expression in the proximal and distal renal tubules, with its extracellular portion being cleaved, yielding a soluble variant that exerts biological effects within the circulation [11]. Evidence stemming from animal models suggests that α-klotho may harbor anti-aging attributes, as elevated levels of this protein correlate with extended lifespans and enhanced resilience against age-related ailments [12]. Numerous studies have shown that higher serum α-klotho concentration can reduce the risk of various diseases, such as diabetes [13], heart failure [14], and malignant tumors [15].

## Aims

In the context of aging and frailty, a particularly intriguing question revolves around the conceivable link between α-Klotho levels and the risk of mortality. Some investigations have unveiled an inverse relationship between plasma α-Klotho concentrations and the likelihood of all-cause mortality, suggesting that heightened α-Klotho levels may confer a notable survival advantage [16–18]. However, the precise mechanisms governing this association remain partially understood, and our understanding of how α-klotho relates to specific mortality causes, such as cancer and cardiovascular disease(CVD), within frail populations remains limited. Given that cancer constitutes a significant contributor to global mortality, with its incidence and impact intensifying with advancing age [19], unraveling the interplay between α-klotho, frailty, and cancer-related mortality holds paramount importance. This longitudinal study endeavors to bridge this knowledge gap by exploring the connection between α-klotho levels and both cancer-related and all-cause mortality within a frail population.

## Methods

### Study population

This population-based study utilized data from the National Health and Nutrition Examination Survey (NHANES), an extensive nationwide research initiative administered by the National Center for Health Statistics (NCHS) under the auspices of the Centers for Disease Control and Prevention (CDC). For this particular research, data from five distinct cycles (2007–2016) were integrated to establish the foundation of the present investigation.

Our initial participant pool encompassed all individuals (n = 50,588) from five cycles (2007–2016) of NHANES. Given that our research focused on frail, middle-aged, and elderly subjects, we excluded participants under the age of 40 (n = 31,244) and those assessed as non-frail (n = 15,273), leaving us with 4071 frail, middle-aged and elderly participants. The core focus of our study being the influence of serum Klotho concentration on mortality in frail patients, we further excluded participants lacking serum Klotho concentration data (n = 1460), as well as those missing mortality follow-up information (n = 3). Subsequently, this refined our sample size to a total of 2,608 participants. The study's selection process, detailing our adherence to the inclusion and exclusion criteria, is depicted in Supplementary Figure S1. The protocol for the NHANES was approved by the National Center for Health Statistics Research Ethics Review Board.

Frailty was assessed using the standard procedure introduced by Searle et al. [20]. The frailty index consisted of 49 deficits covering cognition, dependence, depression, comorbidities, hospital utilization and general health, physical performance and anthropometry, and laboratory values. The frailty index value represents the number of acquired deficits divided by the total potential deficits. Supplementary Table 1 in the Appendix offers a comprehensive outline of the variables within the frailty index and their respective scores. Participants with frailty index values of 0.25 or higher were considered frail and thus included in our study.

### Measurement of serum α-klotho level

Blood α-klotho samples, preserved at − 80 °C, were procured from NHANES participants aged between 40 and 79 years during the cycles spanning from 2007 to 2016. NHANES researchers used a commercially sourced ELISA kit from IBL International, Japan, to obtain these samples, which has a lower limit of detection positioned at 6.15 pg/mL. Duplicate testing of the same set of samples, performed over four distinct days, produced an inter-assay coefficient of variation (CV) of 3.8% and 3.4% respectively for human samples. Each sample was measured in duplicate, and the mean value was employed in calculating the final concentration. Two quality control samples (low and high α-klotho concentrations) were analyzed in duplicate on each ELISA plate. The resultant data was automatically forwarded to the Oracle Management System in the laboratory and scrutinized by the area supervisor. Samples that demonstrated a greater than 10% difference in duplicate results were earmarked for re-analysis. In scenarios where the value for a quality control sample deviated by more than two standard deviations from the assigned value, the entire analytical run was rejected, prompting a repetition of the sample analyses. The mean blood α-klotho concentration of the participants in 5 cycles was 829.20 ± 329.02 pg/ml and more data for each cycle are presented in Supplementary Table 2.

### Ascertainment of mortality

Ascertaining the survivorship of the population under follow-up was carried out using the publicly available NHANES Linked Mortality file, updated as of December 31, 2019. This file is linked with NCHS using a probability-based matching algorithm synchronized with the National Death Index (NDI). NHANES participants were followed until either their recorded death or until December 31, 2019. Instances of participant dislocation, such as relocation or movement out of the surveyed area, leading to an inability to maintain follow-up are considered as missing data. Furthermore, causes of death classifications were implemented through the International Statistical Classification of Diseases, 10th Revision (ICD-10). The mortality outcomes primarily investigated in our study comprised all-cause mortality, cardiovascular disease (CVD)-specific mortality (codes I00-I09, I11, I13, and I20-I51), cancer-specific mortality (codes C00-C97), along with mortality attributed to other causes. For information on linkage methods and further details, refer to the Linked Mortality File.

### Assessment of covariates

The study incorporated established confounding variables from prior research [21]. These adjusted covariates included: survey cycle, age, gender (categorized as male or female), race (white or non-white), education level (high education: yes or no), marital status (stratified as married/cohabiting or widowed/divorced/separated/never married), family income to poverty ratio (designated as PIR, with categories < 1.3, 1.3–3.5, or ≥ 3.5), body mass index (BMI, groups: < 25, 25–30, ≥ 30 kg/m^2^), physical activity (specified as < 150 MET-min/week, 150–960 MET-min/week, 961–1800 MET-min/week, or > 1800 MET-min/week), smoking status (defined as never, past, or current), alcohol consumption (yes or no), serum cotinine, self-reported hypertension and diabetes (each specified as yes or no).

### Statistical analysis

Statistical analysis employed T-test, Mann–Whitney U test for continuous variables, and chi-square tests for categorical variables. Baseline information for study participants was presented using mean ± standard deviation (SD) for continuous variables and proportions for categorical variables. Given the observed gender-specific differences in α-klotho concentrations, quartiles were separately calculated for males and females, using the smallest quartile as a reference. α-klothoHazard Ratios (HRs) with corresponding 95% Confidence Intervals (CIs) were calculated to compare quartiles 2–4 against quartile 1. α-klotho.

Survival analysis was visually presented through Kaplan–Meier curves. Multivariate Cox regression models were employed to assess the impact of α-klotho on survival. Three distinct models were established: Model 1, an unadjusted, crude model; Model 2, adjusted for survey cycles, age, sex, race, PIR, educational attainment, and marriage; and Model 3, which incorporated additional adjustments for BMI, physical activity, smoke status, alcohol intake, serum cotinine, diabetes, and hypertension.

Additionally, an additive Cox proportional hazard model was employed for smoothed curve fitting to analyze the relationship between serum α-klotho concentration (log2-transformed) and mortality risk, with controlling for all previously stated covariates. Log2 transformation of serum α-klotho concentrations was implemented to ensure a normal distribution for improved curve fitting, following the experience of prior research [22]. If the relationship was nonlinear, a threshold effect analysis is performed, which implies that we utilized two-piecewise Cox proportional risk model on both sides of the inflection point to investigate the association between serum α-klotho concentration and the risk of all-cause mortality and cancer-related mortality. Stratified analyses were performed based on sex, age brackets (40–60 years and ≥ 60 years), BMI categories (< 25.00, 25–30 or ≥ 30.00 kg/m^2^), physical activity levels (< 150, 150–960, 961–1800, or > 1800 MET-min/week), as well as the presence of hypertension and diabetes.

The missing covariate data was addressed using the MissForest technique, facilitated by the missForest R package [23]. This technique, built on the powerful Random Forest algorithm, offers a multiple imputation approach particularly adept at handling structurally complex datasets. To assure the robustness of our research, sensitivity analyses were conducted excluding the individuals for whom multiple imputations had been applied. Statistical significance was defined as p < 0.05, with all analyses conducted using R (version 4.2.1) and EmpowerStats (version 4.1).

## Results

### Baseline characteristics of study participants

Table 1 outlines the baseline characteristics of participants, categorized by sex-specific-klotho quartiles. Our study included a total of 2,608 individuals with frailty, among whom 59.89% were female, and the average age was 60.78 ± 10.48 years. The weighted mean α-klotho level was 829.20 ± 329.02 pg/ml. Over a median follow-up period of 83.27 months, the incidence of all-cause mortality was 25.35%. Notably, participants in higher quartiles (Q2-Q4) were more likely to be non-white, had a greater proportion of females compared to quartile 1(Q1), and reported lower levels of physical activity. The recorded blood α-klotho concentrations for males and females were 802.16 pg/ml and 847.32 pg/ml, respectively. Additionally, the blood α-klotho concentrations were quantified across various age brackets and BMI categories, as illustrated in Supplementary Fig. 2.

**Table 1:** Baseline characteristics of middle-aged and older individuals with frailty according to sex-specific serum klotho quartiles

Characteristics	Total population	Sex-specific serum klotho quartile				*P* value
		Q1	Q2	Q3	Q4	
*N*	2608	653	650	651	654	
Age, mean ± SD, years	60.78 ± 10.48	62.07 ± 10.73	61.10 ± 10.19	60.24 ± 10.48	59.73 ± 10.39	< 0.001
Klotho, mean ± SD, pg/ml	829.20 ± 329.02	509.63 ± 85.93	697.07 ± 46.74	860.18 ± 60.45	1248.79 ± 349.59	< 0.001
Sex, *n* (%)						1.000
Male	1046 (40.11%)	262 (40.12%)	261 (40.15%)	261 (40.09%)	262 (40.06%)	
Female	1562 (59.89%)	391 (59.88%)	389 (59.85%)	390 (59.91%)	392 (59.94%)	
Race, *n* (%)						< 0.001
White	1049 (40.22%)	273 (41.81%)	272 (41.85%)	282 (43.32%)	222 (33.94%)	
Non-White	1559 (59.78%)	380 (58.19%)	378 (58.15%)	369 (56.68%)	432 (66.06%)	
High education, *n* (%)						0.633
No	1652 (63.34%)	402 (61.56%)	418 (64.31%)	421 (64.67%)	411 (62.84%)	
Yes	956 (36.66%)	251 (38.44%)	232 (35.69%)	230 (35.33%)	243 (37.16%)	
Marital status, *n* (%)						0.668
Married/cohabiting	1330 (51.00%)	326 (49.92%)	345 (53.08%)	328 (50.38%)	331 (50.61%)	
Widowed/divorced/separated/never married	1278 (49.00%)	327 (50.08%)	305 (46.92%)	323 (49.62%)	323 (49.39%)	
Family income to poverty ratio, *n* (%)						0.595
< 1.3	1254 (48.08%)	310 (47.47%)	330 (50.77%)	309 (47.47%)	305 (46.64%)	
1.3–3.5	1014 (38.88%)	260 (39.82%)	234 (36.00%)	251 (38.56%)	269 (41.13%)	
≥ 3.5	340 (13.04%)	83 (12.71%)	86 (13.23%)	91 (13.98%)	80 (12.23%)	
BMI, *n* (%)						0.643
< 25	389 (14.92%)	95 (14.55%)	102 (15.69%)	89 (13.67%)	103 (15.75%)	
25 ≤ BMI < 30	703 (26.96%)	183 (28.02%)	164 (25.23%)	170 (26.11%)	186 (28.44%)	
≥ 30	1516 (58.13%)	375 (57.43%)	384 (59.08%)	392 (60.22%)	365 (55.81%)	
Physical activity, *n* (%)						0.727
< 150 MET-min/week	1403 (53.80%)	353 (54.06%)	355 (54.62%)	342 (52.53%)	353 (53.98%)	
150–960 MET-min/week	507 (19.44%)	133 (20.37%)	118 (18.15%)	128 (19.66%)	128 (19.57%)	
961-1800MET-min/week	174 (6.67%)	46 (7.04%)	46 (7.08%)	35 (5.38%)	47 (7.19%)	
> 1800 MET-min/week	524 (20.09%)	121 (18.53%)	131 (20.15%)	146 (22.43%)	126 (19.27%)	
Smoke status, *n* (%)						0.52
Now	666 (25.54%)	177 (27.11%)	175 (26.92%)	167 (25.65%)	147 (22.48%)	
Ever	97 (3.72%)	26 (3.98%)	23 (3.54%)	22 (3.38%)	26 (3.98%)	
Never	1845 (70.74%)	450 (68.91%)	452 (69.54%)	462 (70.97%)	481 (73.55%)	
Alcohol intake, *n* (%)						0.016
No	1687 (64.69%)	446 (68.30%)	413 (63.54%)	433 (66.51%)	395 (60.40%)	
Yes	921 (35.31%)	207 (31.70%)	237 (36.46%)	218 (33.49%)	259 (39.60%)	
Hypertension, *n* (%)						0.035
No	1971 (75.58%)	520 (79.63%)	490 (75.38%)	477 (73.27%)	484 (74.01%)	
Yes	637 (24.42%)	133 (20.37%)	160 (24.62%)	174 (26.73%)	170 (25.99%)	
Diabetes, *n* (%)						0.096
No	1090 (41.79%)	305 (46.71%)	261 (40.15%)	251 (38.56%)	273 (41.74%)	
Yes	1518 (58.21%)	348 (53.29%)	389 (59.85%)	400 (61.44%)	381 (58.26%)	
Survival status, *n* (%)						< 0.001
Alive	1947 (74.65%)	438 (67.08%)	509 (78.31%)	507 (77.88%)	493 (75.38%)	
Death	661 (25.35%)	215 (32.92%)	141 (21.69%)	144 (22.12%)	161 (24.62%)	
CVD-related mortality, *n* (%)						< 0.001
Alive	2440 (93.56%)	587 (89.89%)	609 (93.69%)	621 (95.39%)	623 (95.26%)	
Death	168 (6.44%)	66 (10.11%)	41 (6.31%)	30 (4.61%)	31 (4.74%)	
Cancer-related mortality, *n* (%)						0.246
Alive	2468 (94.63%)	611 (93.57%)	616 (94.77%)	625 (96.01%)	616 (94.19%)	
Death	140 (5.37%)	42 (6.43%)	34 (5.23%)	26 (3.99%)	38 (5.81%)	

*PIR* family income to poverty ratio, *BMI* body mass index, *MET* metabolic equivalent

Klotho quartile for male: Q1:181.7–606.95 Q2:606.95–750.1 Q3:750.1–927.35 Q4: 927.35–5038.3

Klotho quartile for female: Q1:152.5–631.5 Q2:631.6–786.7 Q3:786.7–985.8 985.8–4517.8

### Association between α-klotho and mortality

**Fig. 1:**
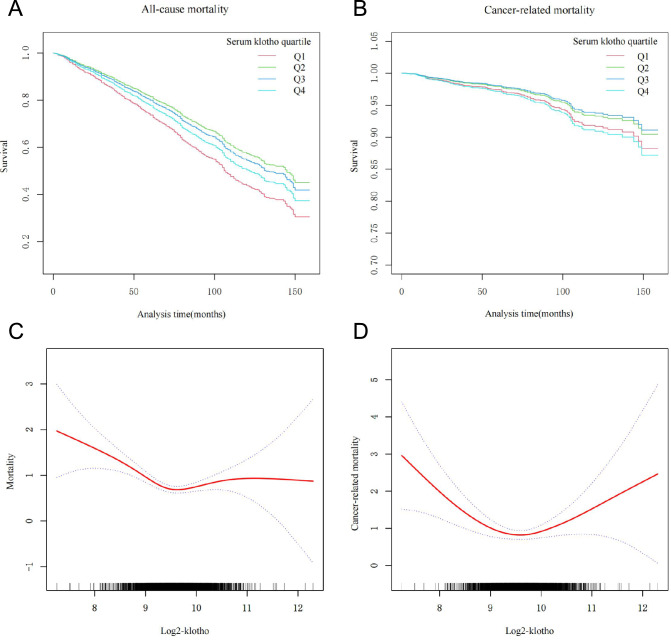
The Cox survival function illustrates the relationship between serum klotho quartiles and all-cause **A** and cancer-related **B** mortality. Smooth curve fitting shows the association between klotho and all-cause **C** and cancer-related **D** mortality, with adjustments for survey cycles, age, sex, race, PIR, education, marriage, BMI, physical activity, smoke status, alcohol intake, serum cotinine, diabetes, and hypertension. The red line represents the mortality risk, and the blue dotted lines indicate the 95% confidence interval

Table 2 presents the association between α-klotho levels and various mortality outcomes, including all-cause mortality, CVD-related mortality, and cancer-related mortality. In the unadjusted Cox regression model, individuals in the highest quartile (Q4) exhibited a significantly lower mortality rate (HR = 0.64, 95% CI 0.52 to 0.78, P for trend < 0.001). After comprehensive adjustment for covariates, these results remained robust and statistically significant (HR = 0.79, 95% CI 0.64–0.97, P for trend = 0.03). This significant association was consistently observed for CVD mortality (HR = 0.48, 95% CI 0.32–0.74, P for trend < 0.001). However, similar findings were not observed in the case of cancer mortality (HR = 0.84, 95%CI 0.54–1.32, P for trend = 0.406). Furthermore, adjusted Kaplan–Meier analysis demonstrated that individuals in the higher quartiles (Q2 or Q3) of klotho experienced a lower cumulative incidence of all-cause mortality and cancer-related mortality throughout the follow-up period when compared to their counterparts in other quartiles (Fig. 1A and B). Similar trends were also noted in the context of cardiovascular disease-related mortality (Figure S3A).

**Table 2:** Associations of serum klotho concentration with all-cause and cancer-related mortality in middle-aged and older individuals with frailty

Exposure	Non-adjusted	*P* for trend	Adjust I	*P *for trend	Adjust II	*P* for trend
Serum Klotho concentration						
All-cause		< 0.0001		0.0312		0.0003
Q1	Reference		Reference		Reference	
Q2	0.61 (0.49, 0.75)		0.64 (0.52, 0.80)		0.64 (0.52, 0.79)	
Q3	0.61 (0.50, 0.75)		0.71 (0.58, 0.88)		0.66 (0.54, 0.81)	
Q4	0.64 (0.52, 0.78)		0.79 (0.64, 0.97)		0.68 (0.55, 0.83)	
CVD-related		< 0.0001		0.0009		0.0001
Q1	Reference		Reference		Reference	
Q2	0.59 (0.40, 0.88)		0.66 (0.44, 0.97)		0.63 (0.43, 0.94)	
Q3	0.41 (0.27, 0.64)		0.49 (0.32, 0.75)		0.44 (0.29, 0.69)	
Q4	0.43 (0.28, 0.67)		0.53 (0.35, 0.82)		0.48 (0.32, 0.74)	
Cancer-related		0.3116		0.8559		0.406
Q1	Reference		Reference		Reference	
Q2	0.73 (0.46, 1.16)		0.81 (0.51, 1.28)		0.75 (0.47, 1.19)	
Q3	0.63 (0.39, 1.01)		0.81 (0.49, 1.26)		0.65 (0.41, 1.05)	
Q4	0.81 (0.52, 1.26)		1.06 (0.68, 1.65)		0.84 (0.54, 1.32)	

*CVD* cardiovascular disease

Model 1: crude model

Model 2: adjusted for survey cycles, age, sex, race, PIR, high education and marriage; Model 3: adjusted for model 2 variables plus BMI, physical activity, smoke status, alcohol intake, serum cotinine, diabetes, hypertension

Klotho quartile for male: Q1:181.7–606.95 Q2:606.95–750.1 Q3:750.1–927.35 Q4: 927.35–5038.3

Klotho quartile for female: Q1:152.5–631.5 Q2:631.6–786.7 Q3:786.7–985.8 985.8–4517.8

### Nonlinear relationship between serum α-klotho concentration and mortality and threshold effect analysis

Upon rigorous adjustment for multiple potential confounding factors, our analysis unveiled a U-shaped nonlinear correlation between log2-klotho levels and both all-cause mortality and cancer mortality (Fig. 1C and D). However, this U-shaped relationship was not observed in the context of CVD mortality (Figure S3B). Building upon these findings, we performed a threshold effect analysis to further validate this U-shaped nonlinear association between log2-klotho and mortality. Our results delineated specific thresholds: the lowest log2-klotho values associated with increased risks of all-cause mortality and cancer-related mortality were identified as 9.48 and 9.55, respectively. These values corresponded to serum α-klotho thresholds of 714.8 and 750.6 pg/ml, respectively (Table 3).

**Table 3:** Threshold effect analysis of log2-klotho on all-cause and cancer-related mortality in frail patients

	Adjusted HR (95% CI)	*P* value
All-cause mortality		
Fitting by the standard linear model	0.82 (0.70, 0.96)	0.0126
Fitting by the two-piecewise linear model		
Inflection point	9.48	
log2-Klotho < 9.48	0.49 (0.37, 0.63)	< 0.0001
log2-Klotho ≥ 9.48	1.30 (1.02, 1.66)	0.036
P for log-likelihood ratio	< 0.001	
Cancer-related mortality		
Fitting by the standard linear model	0.91 (0.65, 1.27)	0.5776
Fitting by the two-piecewise linear model		
Inflection point	9.55	
log2-Klotho < 9.55	0.41 (0.25, 0.69)	0.0008
log2-Klotho ≥ 9.55	1.99 (1.19, 3.35)	0.0093
P for log-likelihood ratio	< 0.001	

Adjusted for survey cycles, age, sex, race, PIR, education and marriage, BMI, physical activity, smoke status, alcohol intake, serum cotinine, diabetes, and hypertension

### Subgroup analyses and sensitivity analyses

**Fig. 2:**
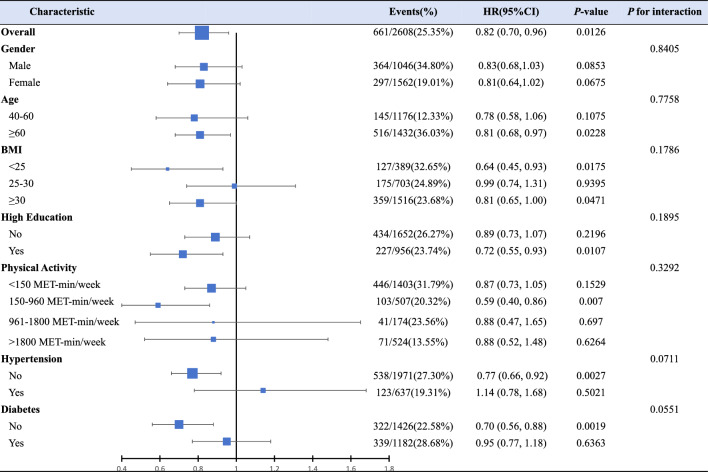
Forest plots present stratified analyses of serum klotho levels and all-cause mortality, with adjustments for survey cycles, age, sex, race, PIR, education, marriage, BMI, physical activity, smoke status, alcohol intake, serum cotinine, diabetes, and hypertension

Our study conducted comprehensive subgroup analyses to investigate the potential influence of demographic characteristics and comorbidities on the association between α-klotho and all-cause mortality (Fig. 2). Subgroup stratification by gender, age, BMI, higher education, physical activity, and hypertension diabetes consistently yielded results with no significant interactions (all P for interaction > 0.05).

Furthermore, sensitivity analyses, which excluded participants whose covariates using multiple interpolation, indicated minimal alterations to our findings. Smooth curve fitting further underscored the U-shaped nonlinear association between log2-klotho and all-cause mortality as well as cancer-related mortality in the frail population (Figure S4 C & D). Importantly, the Kaplan–Meier survival curve analysis mirrored results from the general population analysis (Figure S4 A and B), affirming the robustness of our study's outcomes.

## Discussion

In this population-based longitudinal study, we unveil a U-shaped non-linear relationship between serum α-klotho levels and all-cause mortality among frail individuals. Both high and low serum α-klotho levels appear to elevate the risk of death, while α-klotho levels exhibit a negative linear association with CVD-related mortality. Specifically, individuals with log2-klotho levels of 9.48 (equivalent to 714.8 pg/ml) and 9.55 (equivalent to 750.6 pg/ml) demonstrate the most favorable outcomes in terms of all-cause and cancer-related mortality, respectively. Notably, these findings persist across both unadjusted and multivariate-adjusted analyses, underscoring the potential protective role of elevated α-lotho levels in mitigating mortality risk in frail individuals.

Serum α-klotho levels hold a close association with the onset of frailty in middle-aged and elderly individuals, potentially serving as a biomarker for this clinical syndrome. A systematic review of 16 studies highlights the inverse correlation between α-klotho and physical frailty [24]. Guan et al., in a cross-sectional study employing NHANES data from 2007 to 2016, corroborate this conclusion [25], aligning with Shardell et al.'s results [26]. Given that frailty, intricately intertwined with aging, manifests a soaring prevalence with advancing age, α-klotho, as a therapeutic target against accelerated aging [27], exhibits regulatory prowess over phosphate homeostasis [28], aging-related signaling pathways [29], oxidative stress reduction [30], and fibrosis protection [31]. These attributes collectively contribute to the attenuation of premature mortality risk among frail individuals.

Furthermore, a burgeoning body of research underscores α-klotho's potential therapeutic role within the realm of cancer biology [15, 32, 33]. Αlpha-klotho's anti-fibrotic effects across various tissues present a promising avenue [34]. Age-associated fibrosis, a common pathological hallmark, contributes to organ function decline. Αlpha-klotho effectively curtails the transforming growth factor-beta 1 (TGF-β1) signaling pathway, a pivotal driver of fibrosis [35, 36]. This inhibition acts as a barrier against excessive extracellular matrix deposition and consequent tissue fibrosis [37].

Nevertheless, prior investigations concerning the nexus between serum α-klotho levels and all-cause and cancer-related mortality have yielded incongruent outcomes. Qiao et al.'s research showcases a negative association between serum α-klotho and cancer, but fails to establish a link with cancer-related mortality [38]. Conversely, Chuang et al. discern a non-linear association between soluble alpha-Klotho and all-cause mortality in middle-aged and older Americans, with individuals at the extremities facing heightened mortality risk [39]. In consonance with Chuang et al.'s discoveries, our study elucidates a U shaped correlation between serum α-klotho levels and all-cause mortality as well as cancer mortality. These discrepancies may stem from frail populations' heightened sensitivity to shifts in serum α-klotho levels, leading to increased adverse outcomes, such as mortality. Additionally, α-klotho exists in three subtypes: membrane α-klotho, secreted α-klotho, and soluble α-klotho [11], with immunostaining-based detection potentially susceptible to cross-reactivity [40]. This could result in reduced specificity and measurement biases, offering a plausible explanation for the discordant findings observed in previous investigations.

The implications of our study are of substantial clinical significance. They accentuate the imperative nature of monitoring and optimizing serum α-klotho levels within a specific range, particularly among frail individuals. This optimization holds the potential to curtail the risks associated with all-cause, cancer-related, and CVD-related mortality. Nevertheless, our study possesses certain limitations. Serum alpha-Klotho measurements relied on frozen stored residual serum, potentially introducing measurement biases. Despite adjusting for numerous potential confounders, including sociodemographic factors and comorbidities, reliance on self-reported covariates may have impacted measurement accuracy. Moreover, our observational study design precludes the determination of true causality. Future research endeavors should delve deeper into comprehending the intricate interplay between serum α-klotho levels, frailty, and mortality. Investigating the mechanisms underpinning these associations, particularly in the context of cancer-related mortality, represents a paramount avenue for further exploration. Additionally, interventional studies aimed at modulating serum α-klotho levels may offer innovative strategies for enhancing the longevity and quality of life of frail individuals.

## Conclusion

In summary, our population-based longitudinal investigation unveils the complex interplay between serum α-klotho levels and mortality outcomes in the realm of frail populations. The U-shaped non-linear associations, discerned serum α-klotho thresholds, and varying influences on all-cause and cancer-related mortality risks accentuate the multifaceted functions of α-klotho within the landscape of frailty. These revelations chart a course towards tailored interventions and encourage further exploration into the therapeutic prospects of serum α-klotho modulation, aiming to enhance the overall well-being of middle-aged and older individuals grappling with frailty.

### Supplementary Information

Below is the link to the electronic supplementary material.

Supplementary file1 (DOCX 783 KB)

## Data Availability

All data used in this research, including the serum Klotho concentrations, are publicly accessible and can be freely downloaded from the official NHANES website (https://www.cdc.gov/nchs/nhanes/index.htm).
