# Supplementary material for: Serum klotho levels and mortality patterns in frail individuals: unraveling the u-shaped association
Source: Aging Clin Exp Res. 2024 Apr 11;36(1):92. doi: 10.1007/s40520-024-02730-w (PMC11008069; doi:10.1007/s40520-024-02730-w)

Supplementary Material

# Supplementary Tables

## Supplementary Table 1. Variables in the 49-Item Frailty Index and Their Respective Scorings

| Variable | Scoring | | |
| --- | --- | --- | --- |
| Cognition | Yes=1, No=0 |  |  |
| 1. Experience confusion/memory problems | Difficulty=1, No Difficulty=0 |  |  |
| Dependence | Difficulty=1, No Difficulty=0 |  |  |
| 2. Managing money | Difficulty=1, No Difficulty=0 |  |  |
| 3. Stooping, crouching, kneeling | Difficulty=1, No Difficulty=0 |  |  |
| 4. Lifting or carrying | Difficulty=1, No Difficulty=0 |  |  |
| 5. House chore | Difficulty=1, No Difficulty=0 |  |  |
| 6. Preparing meals | Difficulty=1, No Difficulty=0 |  |  |
| 7. Standing up from armless chair | Difficulty=1, No Difficulty=0 |  |  |
| 8. Getting in and out of bed difficulty | Difficulty=1, No Difficulty=0 |  |  |
| 9. Using fork, knife, drinking from cup | Difficulty=1, No Difficulty=0 |  |  |
| 10. Dressing yourself | Difficulty=1, No Difficulty=0 |  |  |
| 11. Standing for long periods difficulty | Difficulty=1, No Difficulty=0 |  |  |
| 12. Grasp/holding small objects | Difficulty=1, No Difficulty=0 |  |  |
| 13. Attending social event | Difficulty=1, No Difficulty=0 |  |  |
| 14. Push or pull large objects | Difficulty=1, No Difficulty=0 |  |  |
| 15. Walking for a quarter mile difficulty | Difficulty=1, No Difficulty=0 |  |  |
| 16. Walking up 10 steps difficulty | Difficulty=1, No Difficulty=0 |  |  |
| Depressive Symptoms |  |  |  |
| 17. Have little interest in doing things | Nearly every day=1, More than half the days=0.66, Several days=0.33, Not at all=0 |  |  |
| 18. Feeling down, depressed, or hopeless | Nearly every day=1, More than half the days=0.66, Several days=0.33, Not at all=0 |  |  |
| 19. Trouble sleeping or sleeping too much | Nearly every day=1, More than half the days=0.66, Several days=0.33, Not at all=0 |  |  |
| 20. Feeling tired or having little energy | Nearly every day=1, More than half the days=0.66, Several days=0.33, Not at all=0 |  |  |
| 21. Poor appetite or overeating | Nearly every day=1, More than half the days=0.66, Several days=0.33, Not at all=0 |  |  |
| 22. Feeling bad about yourself | Nearly every day=1, More than half the days=0.66, Several days=0.33, Not at all=0 |  |  |
| 23. Trouble concentrating on things | Nearly every day=1, More than half the days=0.66, Several days=0.33, Not at all=0 |  |  |
| Comorbidities |  |  |  |
| 24. Arthritis | Yes=1, Suspect=0.5, No=0 |  |  |
| 25. Thyroid problems | Yes=1, Suspect=0.5, No=0 |  |  |
| 26. Chronic bronchitis | Yes=1, Suspect=0.5, No=0 |  |  |
| 27. Cancer | Yes=1, Suspect=0.5, No=0 |  |  |
| 28. Congestive heart failure | Yes=1, Suspect=0.5, No=0 |  |  |
| 29. Coronary heart disease | Yes=1, Suspect=0.5, No=0 |  |  |
| 30. Angina | Yes=1, Suspect=0.5, No=0 |  |  |
| 31. Heart attack | Yes=1, Suspect=0.5, No=0 |  |  |
| 32. Stroke | Yes=1, Suspect=0.5, No=0 |  |  |
| 33. Blood pressure | Yes=1, Suspect=0.5, No=0 |  |  |
| 34. Diabetes | Yes=1, Suspect=0.5, No=0 |  |  |
| 35. weak/failing kidneys | Yes=1, Suspect=0.5, No=0 |  |  |
| 36. Urinary Leakage | Yes=1, Suspect=0.5, No=0 |  |  |
| Hospital Utilization and Access to Care |  |  |  |
| 37. Self-rated health | Fair, poor=1, Excellent, Very good, good=0 |  |  |
| 38. Health now compared with 1 year ago | Worse=1, About the same, better=0 |  |  |
| 39. Overnight hospital patient in past year | Yes=1, No=0 |  |  |
| 40. Frequency of health care use during past year | None=0, 1-5=0.5, More than 5=1 |  |  |
| 41. Number of prescribed medications | None=0, 1-4=0.5, 5 and more=1 |  |  |
| Physical Performance and Anthropometry |  |  |  |
| 42. Body mass index | <18.5,≥30=1 |  |  |
|  | 25-<30=0.5 |  |  |
|  | 18.5-25=0 |  |  |
| 43. Handgrip strength | MALE: |  | FAMELE: |
|  | For BMI≤24，GS≤29 |  | For BMI≤23，GS≤17 |
|  | For BMI24.1-28,GS≤30 |  | For BMI23.1-26,GS≤17.3 |
|  | For BMI＞28,GS≤32=1 |  | For BMI26.1-29,GS＜18 |
|  |  |  | For BMI＞29,GS≤21=1 |
| Laboratory Values |  |  |  |
| 44. Glycohemoglobin (%) | 0%-5.7%=0, >5.7%=1 |  |  |
| 45. Red blood cell count (million cells/mL) | M: 4.7-6.1=0, Other=1 |  | F: 4.2-5.4=0, Other=1 |
| 46. Hemoglobin (g/dL) | M: 13.5-18=0, Other=1 |  | F: 12-16=0, Other=1 |
| 47. Red cell distribution width (%) | 11.6-14.6=0, Other=1 |  |  |
| 48. Lymphocyte percent (%) | 20-40=0,Other=1 |  |  |
| 49. Segmented neutrophils percent (%) | 40-80=0,Other=1 |  | |

BMI, Body mass index; GS, grip strength.

**1.2 Supplementary Table 2.** NHANES five-round blood klotho concentration distribution

|  | **2007-2008** | **2009-2010** | **2011-2012** | **2013-2014** | **2015-2016** |
| --- | --- | --- | --- | --- | --- |
| **N** | 3006 | 2897 | 2457 | 2767 | 2637 |
| **mean** | 851.66 | 849.48 | 890.35 | 857.91 | 827.09 |
| **SD** | 306.53 | 315.99 | 318.11 | 279.95 | 324.87 |
| **Min** | 156.60 | 152.50 | 206.30 | 151.30 | 153.80 |
| **Max** | 3341.00 | 3829.70 | 3456.00 | 2605.10 | 5038.30 |

# Supplementary Figures

## Supplementary Figure 1. Visual Flowchart for Population Screening


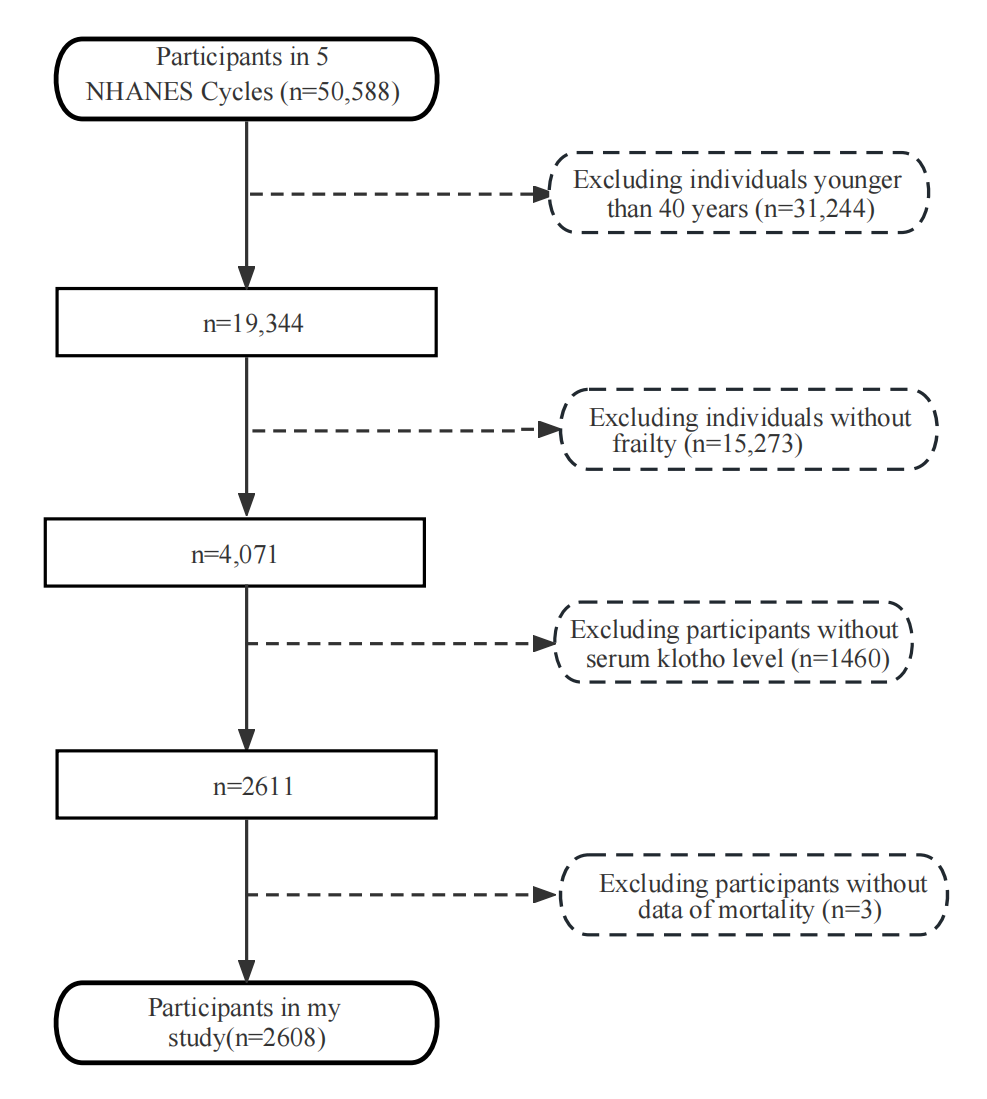


## Supplementary Figure 2. The distribution of blood klotho concentration by gender, age and BMI in this study


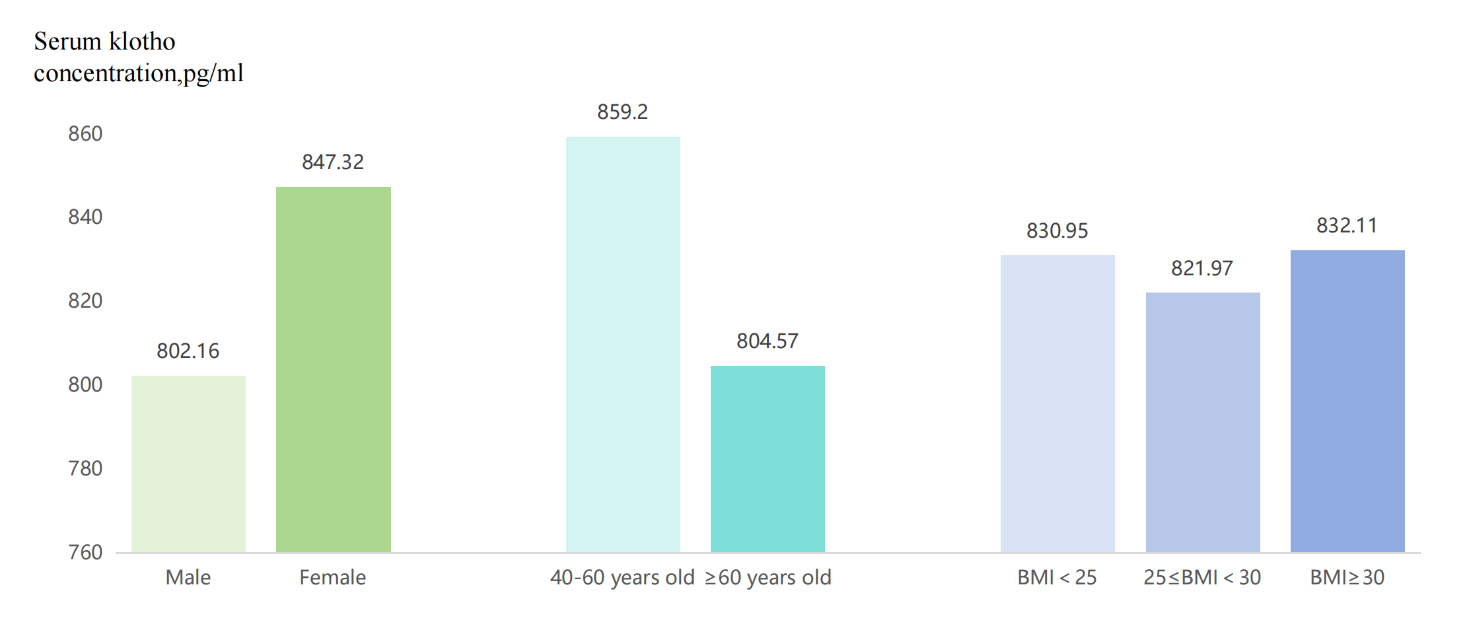


## Supplementary Figure 3. Relationship between cox survival function for cardiovascular disease-related (A) death stratified by sex-specific klotho quartiles. Log2-klotho and cardiovascular disease-related (B) mortality by smooth curve fitting. Adjustment for survey cycles, age, sex, race, PIR, high education, marriage, BMI, physical activity, smoke status, alcohol intake, serum cotinine, diabetes, hypertension.


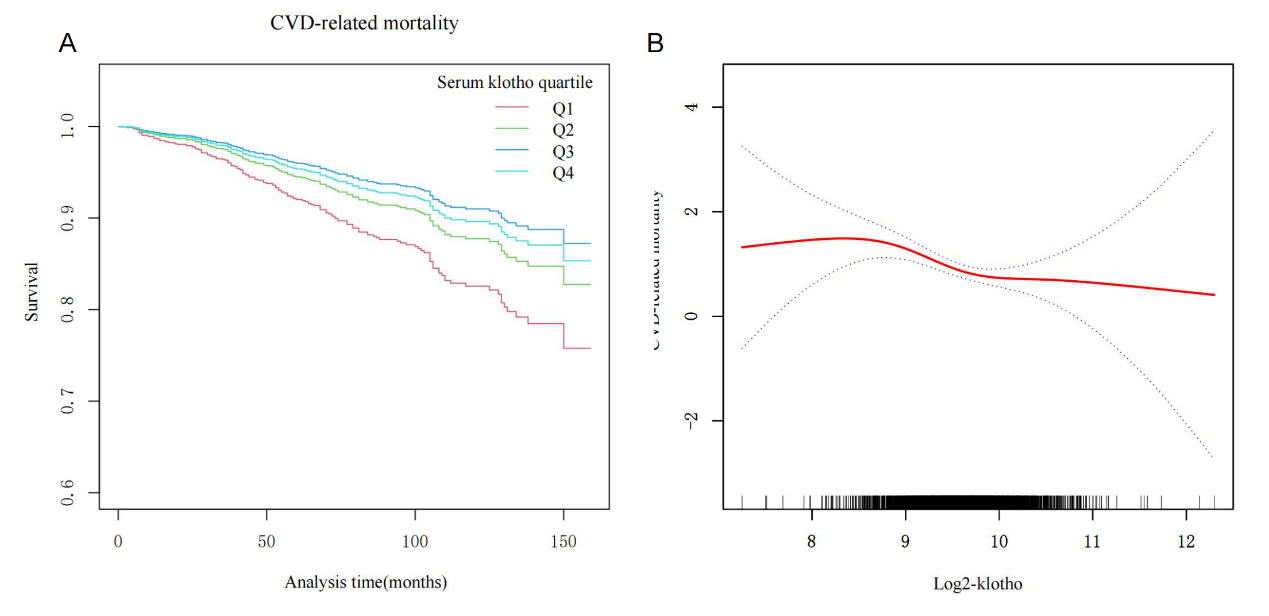


**2.4** **Supplementary Figure 4.** Relationship between cox survival function for all-cause (A), cancer-related (B) and cardiovascular disease-related (C) mortality stratified by sex-specific klotho quartiles in sensitivity analysis excluding participants whose covariates using multiple interpolation. Log2-klotho and all-cause (D), cancer-related (E) and cardiovascular disease-related (F) mortality by smooth curve fitting. Adjustment for survey cycles, age, sex, race, PIR, high education, marriage, BMI, physical activity, smoke status, alcohol intake, serum cotinine, diabetes, hypertension.


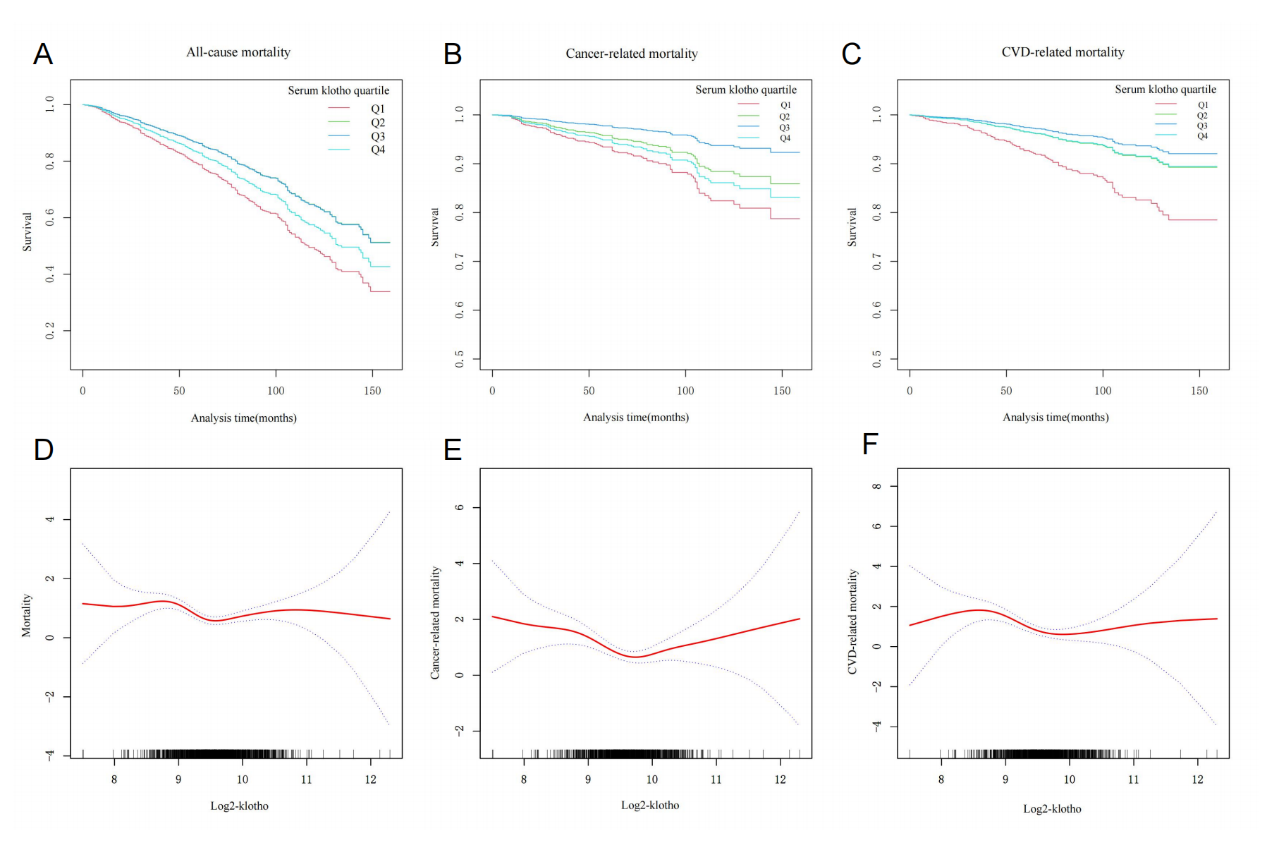

Supplement: Supplementary file 1 — Supplementary file1 (DOCX 783 KB) [file 40520_2024_2730_MOESM1_ESM.docx]
